# Fermentation of *Premna microphylla* Turcz. by *Eurotium cristatum* Enhanced Its Nutrients and Antidiabetic Activity

**DOI:** 10.3390/foods15101632

**Published:** 2026-05-08

**Authors:** Zhengwei Tan, Xiangnan Zhang, Ruzhi Yang, Lei Liu, Yan Zhao, Xingbin Yang

**Affiliations:** 1 Shaanxi Engineering Laboratory for Food Green Processing and Safety Control, and Shaanxi Key Laboratory for Hazard Factors Assessment in Processing and Storage of Agricultural Products, College of Food Engineering and Nutritional Science, Shaanxi Normal University, Xi’an 710119, China; tanzhengwei@snnu.edu.cn; 2 College of Life Sciences, Shaanxi Normal University, Xi’an 710062, China; 18230959671@163.com (R.Y.); 13629120305@163.com (L.L.); yanzhao@snnu.edu.cn (Y.Z.)

**Keywords:** *Eurotium cristatum*, *Premna microphylla* Turcz., fermentation, type 2 diabetes, short-chain fatty acids

## Abstract

This study developed a probiotic *Eurotium cristatum*-fermented *Premna microphylla* Turcz. (EFPT) and systematically evaluated its nutritional profile and antidiabetic effects in a type 2 diabetes mellitus (TIIDM) mouse model. After 8 days of fermentation, the contents of pectin (9.001% vs. 4.222%) and water-soluble polysaccharides (13.339% vs. 4.826%) increased significantly (*p* < 0.05), whereas the levels of polyphenols (12.12% vs. 15.917%) and soluble proteins (3.829% vs. 5.797%) decreased (*p* < 0.05). Notable alterations were also observed in short-chain fatty acids (SCFAs), monosaccharide composition, and amino acid profiles. Specifically, the levels of propionic acid, histidine, threonine, serine, glycine, lysine, glutamic acid, and methionine decreased significantly (*p* < 0.05), while those of butyric acid, valeric acid, galactose, valine, alanine, and proline increased (*p* < 0.05). Furthermore, EFPT exhibited dose-dependent antidiabetic effects and showed greater efficacy than non-fermented *Premna microphylla* Turcz. powder in high-fat diet combined with streptozotocin-induced TIIDM mice (*p* < 0.05). These effects were primarily associated with enhanced SCFAs production and the amelioration of liver and kidney damage. Overall, these findings suggest that *Eurotium cristatum* fermentation enhances the bioactive properties of *Premna microphylla* Turcz., contributing to its improved nutritional quality and potent antidiabetic activity.

## 1. Introduction

Rapid changes in lifestyle and dietary patterns have contributed to a continuous increase in the incidence of chronic metabolic disorders, particularly obesity and type 2 diabetes mellitus (TIIDM) [1,2]. TIIDM is primarily characterized by insulin resistance and impaired insulin secretion, leading to persistent hyperglycemia and the subsequent development of severe complications, including cardiovascular, ocular, renal and neurological disorders [3,4,5]. Although the prevention and management of TIIDM largely relies on lifestyle modifications, such as maintaining regular routines, engaging in physical activity, and adhering to a healthy diet, pharmacological interventions are often required for effective glycemic control [6,7]. Commonly prescribed glucose-lowering agents include biguanides, insulin secretagogues and α-glucosidase inhibitors, as well as exogenous insulin administration [8,9]. Despite their proven efficacy, these treatments are often associated with high economic costs, potential side effects, and reduced patient compliance, particularly in the case of long-term insulin therapy [9]. Therefore, increasing attention has been directed toward the development of natural, safe, and cost-effective functional foods or bioactive compounds with antidiabetic properties.

*Premna microphylla* Turcz. (PMT) is primarily distributed in tropical and subtropical regions of the Eastern Hemisphere and is rich in pectin (about 30–40%), proteins (about 15–30%), flavonoids (about 2.5%) and polyphenolic (about 3%) compounds [10]. In China, PMT is predominantly processed into a traditional gel-like food for consumption [10]. Previous studies have demonstrated that PMT possesses various biological activities, including antioxidant, anti-inflammatory, and anti-obesity effects, suggesting its potential as a functional food ingredient [11,12,13].

Fermentation is a traditional and effective bioprocessing strategy widely used to extend shelf life and improve the nutritional quality, flavor, and functionality of foods [14]. During food fermentation, microorganisms convert insoluble macromolecules into soluble and low-molecular-weight bioactive compounds, thereby enhancing their bioaccessibility and bioactivity [14,15]. For instance, natural fermentation of carrots has been reported to improve their antioxidant and anti-obesity properties [16]. However, whether fermentation can further enhance the nutritional characteristics and bioactivity of PMT remains unclear.

*Eurotium cristatum* (EC), commonly known as the “golden-flower” fungus, is a non-pathogenic probiotic microorganism widely used in fermented foods [17]. During its growth, EC secretes various extracellular enzymes, including pectinases, proteases, cellulases and polyphenol oxidases, which facilitate the degradation of complex macromolecules. For example, insoluble dietary fibers can be converted into water-soluble polysaccharides, while polyphenols may be transformed into bioactive derivatives such as theabrownins [17]. As a result, EC fermentation is often associated with improved nutritional and functional properties of food substrates. Previous studies have shown that EC fermentation of soybeans enhances nutrient composition, flavor, and gut-regulatory effects [18]. Similarly, fermentation of mulberry leaves by EC increased the content of theabrownins and improved α-glucosidase inhibitory activity [19]. It is worth noting that EC-fermented Coptis herbal pairs exhibit enhanced antidiabetic activity [20]. However, to date, no studies have investigated the effects of EC fermentation on the nutritional quality of PMT or its antidiabetic potential.

Therefore, the present study aimed to systematically investigate the effects of EC fermentation on PMT. Specifically, the changes in major nutritional and bioactive components during fermentation were analyzed, and the antidiabetic activity of fermented PMT was evaluated using a TIIDM mouse model induced by a high-fat diet (HFD) combined with streptozotocin (STZ). This study provides a theoretical basis for the development of PMT-based functional foods with enhanced health benefits.

## 2. Materials and Methods

### 2.1. Materials and Reagents

The PMT was sourced from the local supermarket in October 2024 (Xi’an, China). EC strain is numbered CICC 2099 in the China Industrial Microbial Strain Collection. A 1-Phenyl-3-methyl-5-pyrazolone (PMP) and a series of standard monosaccharides including mannose, rhamnose, _D_-galacturonic acid, glucose, galactose, _DL_-arabinose, and six standard short-chain fatty acids (acetic acid, propionic acid, butyric acid, isobutyric acid, valeric acid, and isovaleric acid) were obtained from Sigma-Aldrich (St. Louis, MO, USA). Total cholesterol (TC, A111-1-1), triglycerides (TG, A110-1-1), low-density lipoprotein cholesterol (LDL-C, A113-1-1), high-density lipoprotein cholesterol (HDL-C, A112-1-1), alanine aminotransferase (ALT, C009-2-1), aspartate aminotransferase (AST, C0010-2-1), catalase (CAT, A007-2-1) and glutathione (GSH, A006-2-1) were purchased from Nanjing Jiancheng Bioengineering Institute (Nanjing, China). Other reagents used in this research were of the needed grade and commercially available.

### 2.2. Determination of Optimal Conditions in PMT Fermentation

Determine the optimal fermentation conditions based on our previous research, with appropriate modifications [18]. In detail, after activation and incubation, the concentration of the EC inoculum was adjusted to 1 × 10^6^–10^7^ CFU/mL. The EC fermented PMT power (EFPT) was performed under the following conditions to determine the optimal fermentation parameters: (1) *Solid–liquid ratio*: 26 g PMT powder was mixed with 10.4, 15.6, 20.8, 26, and 31.2 mL of water in 250 mL conical flask to achieve solid–liquid ratios of 1:0.4, 1:0.6, 1:0.8, 1:1.0, and 1:1.2, respectively, and inoculated with a 1.5% inoculum, fermented at 28 °C for 6 days. (2) *Inoculum size*: 26 g PMT powder was mixed with 20.8 mL of water in a 250 mL conical flask; 0.5%, 1.0%, 1.5%, 2.0% and 2.5% inoculum mentioned above were added to the flasks, and the fermentation was conducted at 28 °C for 6 days. (3) *Substrate thickness*: 15, 19, 23, 26 and 29 g PMT powder was mixed with equal weight water in 250 mL conical flasks to achieve substrate thicknesses of 1.0, 1.5, 2.0, 2.5, and 3.0 cm, respectively, and then was sealed, sterilized, and inoculated with a 1.5% inoculum at 28 °C for 6 days. Given that pectin is the primary active ingredient in PMT, the optimal fermentation parameters were determined based on the content of pectin as the evaluation index. All experiments were performed in triplicate to ensure reliability.

Moreover, under the optimized conditions of solid–liquid ratio (1:0.8), inoculum size (2.5%) and substrate thickness (2.5 cm), the dynamic changes in nutritional quality of PMT during EC-based solid-state fermentation time were measured. In brief, 26 g of PMT powder and 20.8 mL of ultrapure water were mixed in 250 mL conical flasks, homogenized, and sterilized. After sterilization, the fermentation substrate was cooled to room temperature and inoculated with a 2.5% inoculum. The samples were then incubated at 28 °C and collected at 0, 2, 4, 6, and 8 days.

### 2.3. Determination of the Dynamic Change in Chemical Components in EFPT

#### 2.3.1. Determination of Pectin

Pectin content was determined by the carbazole–sulfuric acid method, using galacturonic acid as the standard (*y* = 0.0045*x* + 0.15, R^2^ = 0.9989) [21]. Pectin was extracted following our previously described method [22]. Briefly, the fermentation powder was mixed with distilled water (*w*:*v*, 1:30) and the pH adjusted to 1.6 with HCl (1 mol/L). The mixture was incubated in a water bath at 85 °C for 2 h, then centrifuged at 4500 r/min (Velocity 14R, Dynamica, Shanghai, China) and the supernatant was collected. The supernatants were concentrated, and an equal volume of anhydrous ethanol was added; then, the pH was adjusted to 3.5 with 0.10 mol/L HCl, precipitated. Next, ethanol was added to the precipitate (*v*:*w*, 5:1), and the mixture was stirred thoroughly, sedimented, and centrifuged. The precipitate was dialyzed using a cellulose dialysis bag (8 kDa). Specifically, after the precipitation was redissolved in distilled water, a 24 h dialysis was performed using running water, followed by dialysis with distilled water. During the distilled water dialysis process, when the distilled water had been replaced three times, the dialysis was complete. The dialysis solution was frozen in a −80 °C freezer for 24 h, then removed and placed in a pre-cooled freeze-dryer (CTFD-10S, Qingdao Creatrust Electronic Technology Co., Ltd., Qingdao, China) for 48 h of freeze-drying.

#### 2.3.2. Determination of Total Polyphenols and Flavones

Total polyphenols content was determined by the Folin–Ciocalteu method. We thoroughly mixed 200 μL of the sample solution (1 mg/mL) with 800 μL of distilled water and 200 μL of Folin–Ciocalteu reagent, and then stored it in the dark for 6 min. Next, we added 2 mL of 7% Na_2_CO_3_ solution, mixed thoroughly, and allowed it to stand for 90 min before measuring the absorbance at 760 nm using a microplate reader (BioTek Synergy H1, Agilent, Santa Clara, CA, USA), and a standard curve was plotted using gallic acid as the standard (y = 0.072x + 0.0724, R^2^ = 0.9956) [23]. Total flavone content was measured by the NaNO_2_–Al(NO_3_)_3_–NaOH method. We mixed 1 mL of the sample solution (1 mg/mL) with 0.3 mL of 5% NaNO_2_, allowed it to stand for 6 min, then added 0.3 mL of 10% Al(NO_3_)_3_, shook it well, and allowed it to stand for 6 min. Finally, 4.0 mL of 4% NaOH was added, and the absorbance was measured at 510 nm, and a standard curve was plotted using rutin as the standard (*y* = 3.5821*x* + 0.0324, R^2^ = 0.9981) [24].

#### 2.3.3. Evaluation of Short-Chain Fatty Acid Levels

SCFAs were analyzed using GC-MS with an Agilent DB-WAX capillary column (Santa Clara, CA, USA), and the ion source temperature was set to 230 °C. The initial temperature of the capillary column was 90 °C, which was increased at a rate of 10 °C per min to 150 °C, followed by a further increase at a rate of 20 °C per min to 230 °C, where it was held for 3 min [25].

#### 2.3.4. Measurement of Polysaccharides and Monosaccharide Composition

Crude soluble polysaccharides were extracted according to our previous method and detected by the sulfuric acid–phenol method with glucose as the standard (*y* = 0.0185*x* + 0.0694, R^2^ = 0.9970) [26]. Next, monosaccharide composition of crude soluble polysaccharides was analyzed by High-Performance Liquid Chromatography (U3000, Thermo Fisher Scientific, Waltham, MA, USA). Analysis was performed using a C18 column (4.6 mm diameter × 250 mm, 5 μm, Venusil, Tianjing, China). In brief, polysaccharides (20 mg) were hydrolyzed with trifluoroacetic acid (2 mL, 3 mol/L) at 95 °C for 8 h. After the sample was centrifuged and dried, 1 mL of ultrapure water was added to dissolve it. Then, we added 0.3 mL of NaOH (0.3 mol/L) and 0.2 mL of PMP (0.5 mol/L), and maintained it at 70 °C for 1 h for derivatization. Mobile phase A: acetonitrile; mobile phase B: 3.3 mmol/L potassium dihydrogen phosphate, 3.6 mM triethylamine, pH 7.5. The gradient elution procedure was as follows: 95% B, 0–5 min; 95–90% B, 5–8 min; and 90–90% B, 8–30 min, with an injection volume of 10 μL and a flow rate of 1.0 mL/min. The UV detection wavelength was 250 nm, and the separation temperature was 37 °C.

#### 2.3.5. Evaluation of Soluble Proteins and Amino Acid Species

The Bradford method was employed to analyze the total soluble protein content with bovine serum albumin as a standard (*y* = 0.0084*x* + 0.6928, R^2^ = 0.9925). The amino acid composition of EFPT was determined using an automated amino acid analyzer (LA8080, Hitachi, Tokyo, Japan).

### 2.4. Animal Experiment

#### 2.4.1. Preparation of the Aqueous Extracts from the EC-Fermented and -Unfermented PMT

EC-fermented PMT samples obtained under optimal fermentation conditions were thoroughly mixed with purified water (*w*:*v*, 1:20), incubated in a 60 °C water bath for 2 h, and then filtered to collect the supernatant. The supernatant was concentrated and lyophilized to yield the EC-fermented PMT aqueous extract (FT). Similarly, unfermented PMT aqueous extract (T) was prepared in the same process. In brief, the unfermented PMT powder was mixed with pure water (*w*:*v* = 1:20) and incubated in a water bath (60 °C, 2 h). Then, it was filtered to collect the supernatant.

#### 2.4.2. Diabetes Model Mice Construction and Intervention

**Figure 3:**
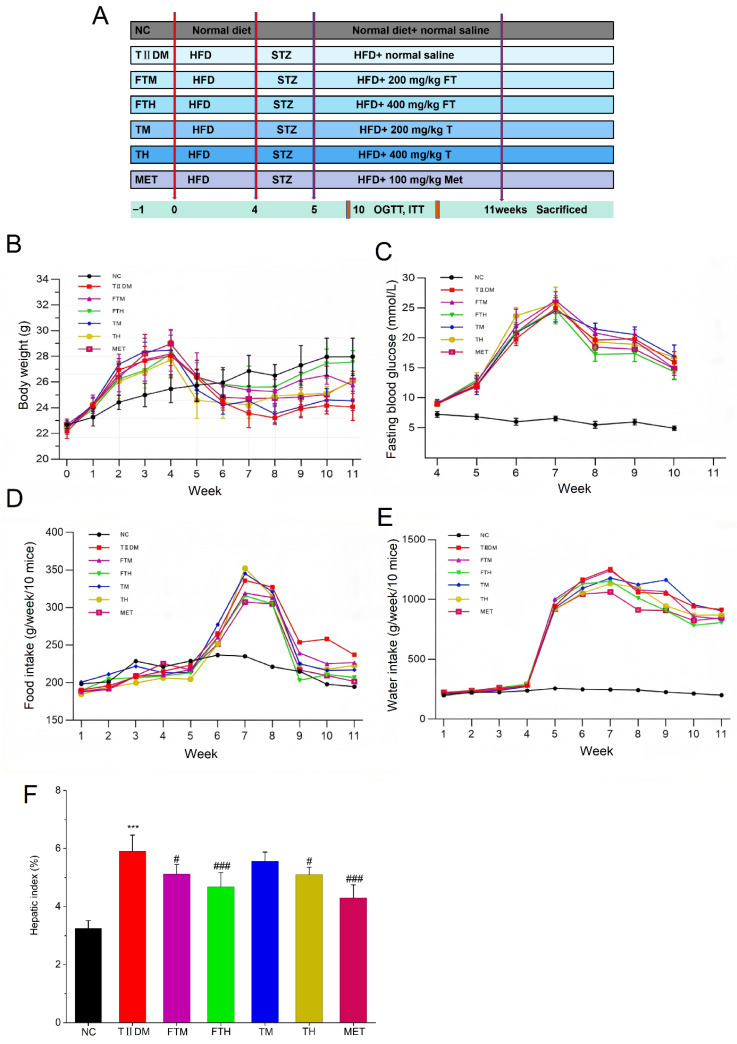
*Premna microphylla* Turcz. fermented by *Eurotium cristatum* significantly ameliorated the symptoms of TIIDM in mice (*n* = 10). (**A**) Experimental design, (**B**) body weight, (**C**) fasting blood glucose, (**D**) food intake, (**E**) water intake, (**F**) hepatic index. (*), (**) and (***) represent *p* < 0.05, *p* < 0.01 and *p* < 0.001 compared with NC mice, and (#), (##) and (###) represent *p* < 0.05, *p* < 0.01 and *p* < 0.001 compared with TIIDM mice, respectively.

A total of 70 healthy male C57BL/6J mice (5 weeks, 20 ± 2 g) were acquired and kept at the Experimental Animal Center of the Shaanxi Normal University (Xi’an, China). The Laboratory Animal Ethics Committee of Shaanxi Normal University reviewed and approved all procedures (protocol code: GZK2025-097; approval date: 14 March 2025). Following 7 days of adaptive feeding, the mice were randomly distributed into 7 groups (Figure 3A): the normal control group (NC, *n* = 10), the model group (TIIDM, *n* = 10), the FT low-dose group (FTM, *n* = 10), the FT high-dose group (FTH, *n* = 10), the T low-dose group (TM, *n* = 10), the T high-dose group (TH, *n* = 10), and the metformin control group (MET, *n* = 10). The NC group received a standard diet. Conversely, mice in the TIIDM, FTM, FTH, TM, TH and MET groups received a high-fat diet (HFD). Following 4 weeks of feeding, mice in the NC group were intraperitoneally injected with citrate buffer (0.1 mol/L, pH 4.5) as a control, while the other groups’ mice were injected intraperitoneally with 50 mg/kg bw streptozotocin (STZ) once daily for three consecutive days to induce diabetes. Using a glucometer to measure the fasting blood glucose levels in mice, and mice with fasting blood glucose levels greater than or equal to 11.1 mmol/L were successfully diagnosed with TIIDM. Normal saline was given orally to the mice in the NC and TIIDM groups, and mice in the FTM (200 mg/kg FT), FTH (400 mg/kg FT), TM (200 mg/kg T) and TH (400 mg/kg T) groups were treated by oral gavage, and the MET group was treated with 100 mg/kg of metformin by oral administration as a positive control. At the end of 6 weeks, mice were sacrificed and serum, liver, kidney, colon, cecum, and pancreas tissues were collected and frozen at −80 °C for subsequent evaluation.

### 2.5. Oral Glucose Tolerance Test and Insulin Tolerance Test

The oral glucose tolerance test (OGTT) and insulin tolerance test (ITT) were assessed according to our previous work [25].

### 2.6. Determination of Biochemical Parameters

Following the manufacturer’s instructions, the levels of TC, TG, LDL-C and HDL-C in serum, ALT and AST in liver, CAT and SOD in kidney, and CAT and GSH in pancreas were determined using a microplate reader (BioTek Synergy H1, Agilent, Santa Clara, CA, USA).

### 2.7. Histopathological Observation

The injury of the liver was assessed by hematoxylin and eosin (H&E) staining.

### 2.8. Data Analysis

The data were statistically analyzed by SPSS software (version 25.0) and expressed as mean ± standard deviation (SD). Comparison among the multiple groups using one-way analysis of variance (ANOVA) followed by Tukey’s post hoc test was performed using PASW Statistics 18. Statistically significant was considered to be *p* < 0.05. Figures were drawn using Origin 2024b.

## 3. Results

### 3.1. Establishment of Optimal Conditions for PMT Fermentation with EC

**Figure 1:**
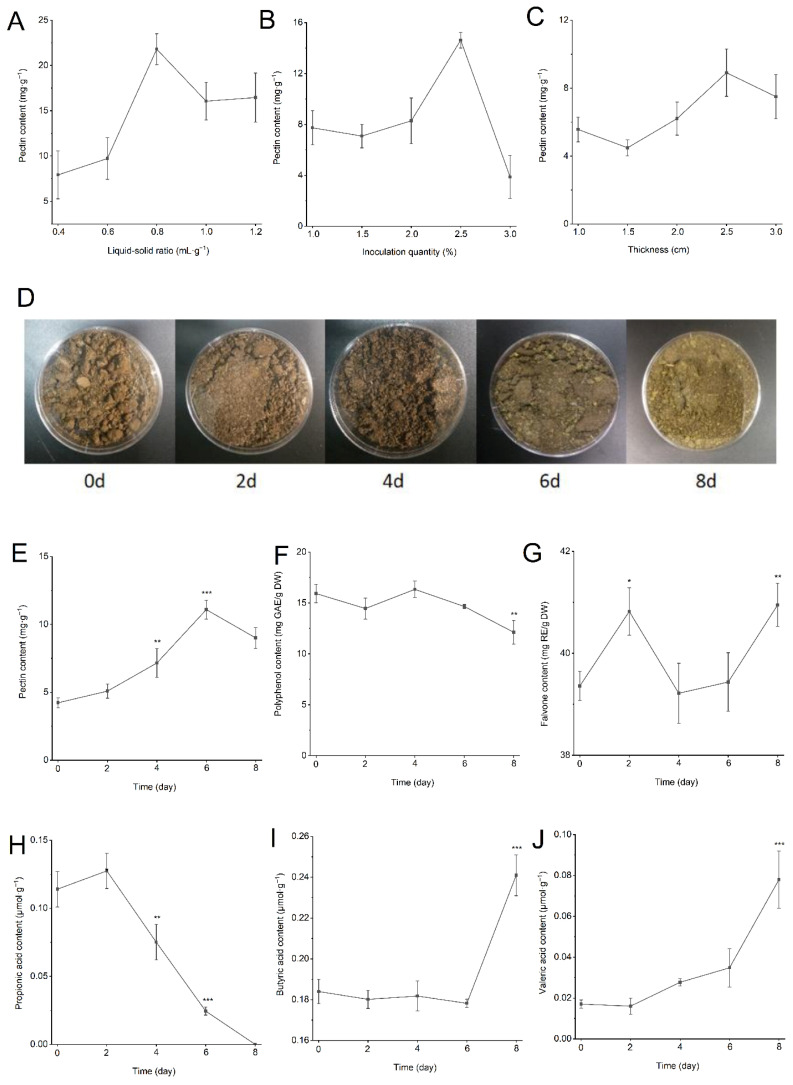
Single-factor and quantitative analysis of the appearance of pectin, polyphenol, falvone, and short-chain fatty acids of *Eurotium cristatum*-fermented *Premna microphylla* Turcz. (EFPT). The effects of (**A**) liquid–solid ratio, (**B**) inoculum amount, (**C**) thickness on the contents of pectin in EFPT. The dynamic changes of (**D**) color, (**E**) pectin, (**F**) total polyphenols, (**G**) falvone, (**H**) propionic acid, (**I**) butyric acid, and (**J**) valeric acid of *Premna microphylla* Turcz. fermented by *Eurotium cristatum* (*n* = 3). (*) *p* < 0.05, (**) *p* < 0.01 and (***) *p* < 0.001 vs. unfermented *Premna microphylla* Turcz.

As shown in Figure 1A, with the increase in the liquid–solid ratio, the pectin content in EFPT gradually rose, reaching its peak at a ratio of 1:0.8 (21.800 ± 1.709 mg·g^−1^), followed by a subsequent decline. As illustrated in Figure 1B, when the inoculum size ranged from 1.0% to 2.5%, the pectin content in EFPT increased alongside the inoculum size, achieving its maximum content at 2.5% (14.623 ± 0.620 mg·g^−1^). Similarly, the pectin content in EFPT exhibited an upward trend with increasing substrate thickness (Figure 1C), reaching the highest level when the thickness was 2.5 cm (8.908 ± 1.392 mg·g^−1^), and then declined.

### 3.2. Dynamic Changes in Nutritional Quality of PMT During EC-Based Solid-State Fermentation

Figure 1D shows the color change in PMT powder fermented by EC over an 8-day period under the optimal conditions. As fermentation progressed, colonies of EC became clearly visible at 6 days in EFPT samples. Subsequently, by day 8, the distribution of EC within the EFPT became more uniform. Additionally, the moisture content of the EFPT samples decreased over time, resulting in a drier consistency of the substrate. The pectin content in EFPT reached its peak (1.086 ± 0.698 mg·g^−1^) on day 6 (*p* < 0.001), followed by a decline on day 8 (Figure 1E). Conversely, during the fermentation process, the polyphenol content in EFPT exhibited a downward trend (Figure 1F), decreasing from 15.917 ± 0.903 mg GAE/g DW in the unfermented samples to 12.120 ± 1.156 mg GAE/g DW after 8 days of fermentation by EC (*p* < 0.01). However, the flavonoid content exhibited a fluctuating trend during fermentation, characterized by an initial increase, followed by a decrease, and a subsequent rise (Figure 1G). The flavonoid content in the unfermented PMT powder was 39.359 ± 0.289 mg RE/g DW, which significantly increased to 40.821 ± 0.464 mg RE/g DW in EFPT after 2 days of fermentation (*p* < 0.05). Nevertheless, a declining trend was observed on day 4, with the concentration falling even below that of the unfermented samples. Subsequently, the content increased from day 6 to day 8, reaching a maximum value of 40.949 ± 0.424 mg RE/g DW on day 8 (*p* < 0.01). Figure 1H–J illustrates the dynamic changes in SCFAs during the fermentation process. Acetic acid remained undetected throughout the entire fermentation period, while the concentration of propionic acid initially increased and subsequently decreased, eventually becoming undetectable by day 8. Notably, butyric acid levels surged on day 8, reaching 0.241 ± 0.010 μmol·g^−1^ (*p* < 0.001). Similarly, the valeric acid content in EFPT peaked at 0.078 ± 0.014 μmol·g^−1^ on day 8 (*p* < 0.001).

**Figure 2:**
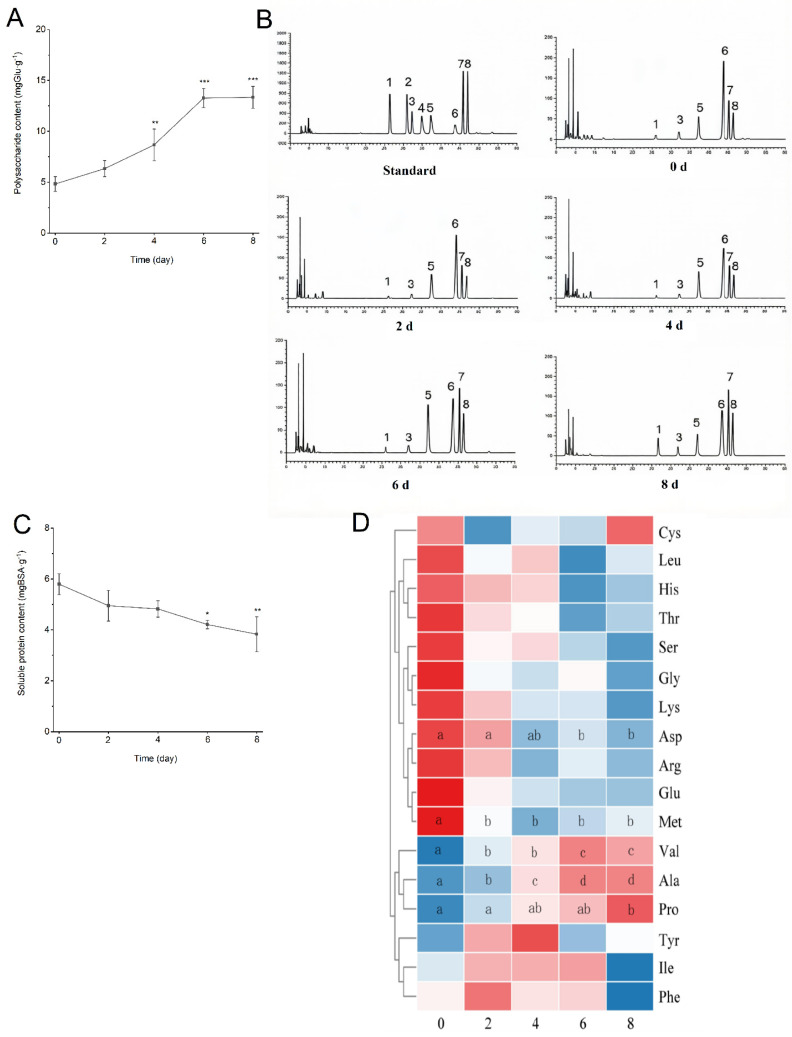
The dynamic changes in total polysaccharides, monosaccharide composition, total protein and amino acids profile in EFPT at different fermentation time points (*n* = 3). (**A**) Total polysaccharides of EFPT. (**B**) Monosaccharide composition chromatogram of EFPT, 1, Mannose; 2, Ribose; 3, Rhamnose; 4, D-Glucuronic acid; 5, D-Galacturonic acid; 6, Glucose; 7, Galactose; 8, Arabinose. (**C**) Total protein and (**D**) amino acids profile of EFPT. (*) *p* < 0.05, (**) *p* < 0.01 and (***) *p* < 0.001 vs. unfermented *Premna microphylla* Turcz., different letters represent significant differences at *p* < 0.05. While the following abbreviations are used in this figure: Cys: Cystine; Leu: Leucine; His: Histidine; Thr: Threonine; Ser: Serine; Gly: Glycine; Lys: Lysine; Asp: Aspartic acid; Arg: Arginine; Glu: Glutamic acid; Met: Methionine; Val: Valine; Ala: Alanine; Pro: Proline; Tyr: Tyrosine; Ile: Isoleucine; Phe: Phenylalanine.

Moreover, Figure 2A illustrates the temporal variations in the polysaccharide content of PMT fermented with EC. Compared to the unfermented PMT powder (4.826 ± 0.729 mg Glu·g^−1^), the soluble polysaccharide content in EFPT increased consistently as the fermentation process progressed, reaching a peak of 13.264 ± 0.943 mg Glu·g^−1^ at day 6 (*p* < 0.001), and subsequently remained stable from day 6 to day 8. As shown in Figure 2B and Table 1, ribose and glucuronic acid were not detected in EFPT throughout the fermentation process. Compared with the EFPT sample at day 0, the mannose content in EFPT decreased to 1.17% at the end of fermentation. Similar trends were observed for rhamnose and arabinose; at the end of fermentation, their contents had increased to 18.84% and 9.63%, respectively, both exceeding their initial levels. In contrast, galacturonic acid level peaked on day 6 (28.70%) and sharply declined by day 8. Glucose remained the predominant monosaccharide, consistently accounting for approximately 40% of the total molar ratio composition.

Figure 2C indicates that the soluble protein content of the EFPT showed an overall downward trend during fermentation with EC. Compared with the unfermented sample, the soluble protein content decreased significantly on day 6 (*p* < 0.05), declining from 5.797 ± 0.415 μg BSA·mL^−1^ to 4.210 ± 0.165 μg BSA·mL^−1^. This decline continued, and the lowest value was recorded on day 8, at 3.829 ± 0.687 μg BSA·mL^−1^. Figure 2D shows that a total of 17 amino acids were detected in EFPT. During fermentation, the contents of five amino acids changed significantly: aspartic acid (Asp), methionine (Met), valine (Val), alanine (Ala), and proline (Pro), respectively. Specifically, Asp and Met decreased upon EC-mediated fermentation of PMT, whereas Val, Ala, and Pro increased. Val exhibited a significant increase between day 0 and day 6 (*p* < 0.05); Ala increased significantly throughout the entire fermentation period (days 0–6, *p* < 0.05); and Pro showed a gradual accumulation, with its increase becoming significant only at day 8 (*p* < 0.05).

### 3.3. Consumption of EC-Fermented PMT Improved HFD/STZ-Induced TIIDM Symptoms

As shown in Figure 3B,C, compared with the NC mice, TIIDM mice exhibited a significant reduction in body weight and elevated blood glucose levels. However, treatment with FT and T mitigated the weight loss and markedly decreased fasting blood glucose levels in the TIIDM mice. Notably, the therapeutic effect in the FTH group was more pronounced than those in the FTM, TH, and TM groups, demonstrating a dose-dependent relationship. Furthermore, while TIIDM mice showed increased food and water intake relative to the NC group, oral administration of FT or T achieved an effect comparable to metformin (Met) in terms of reducing food and water consumption (Figure 3D,E). Compared with the NC group, the liver index of the TIIDM group increased significantly (Figure 3F, *p* < 0.001). Conversely, all intervention groups showed a reduction in liver index compared with the TIIDM group, with the most significant decrease observed in the FTH-treatment group (*p* < 0.001).

### 3.4. Treatment with EC-Fermented PMT Recovered Lipid and Glucose Homeostasis in TIIDM Mice

**Figure 4:**
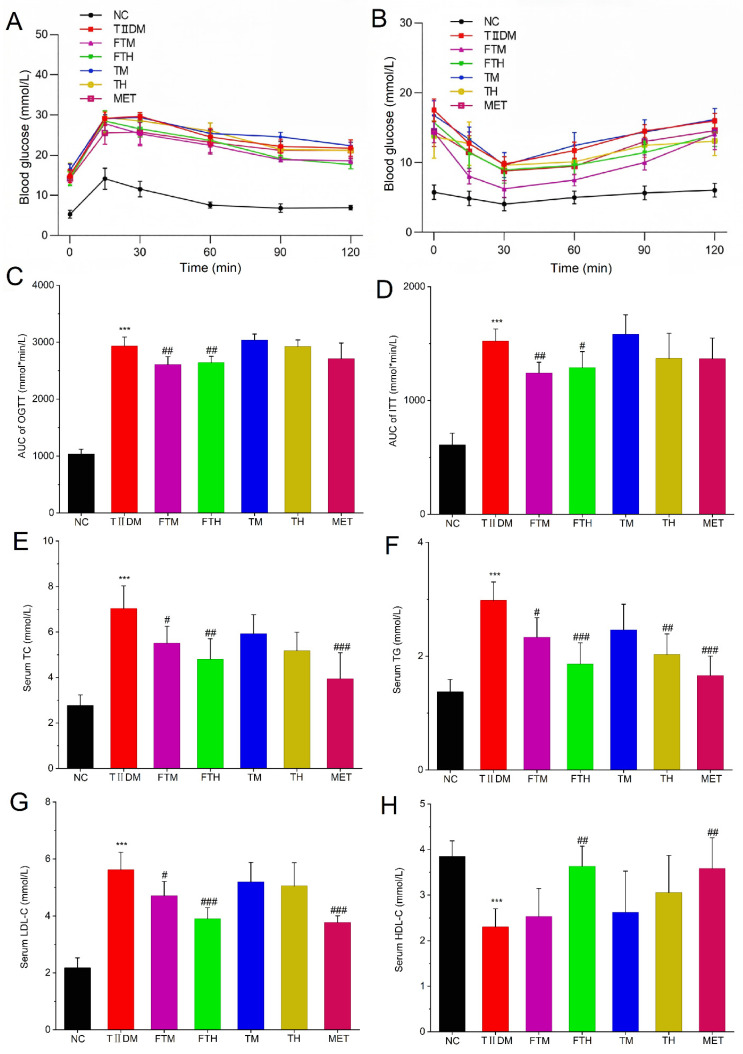
Treatment with *Eurotium cristatum*-fermented *Premna microphylla* Turcz. recovered lipid and glucose homeostasis in TIIDM mice (*n* = 10). (**A**) OGTT, (**B**) ITT, (**C**) AUC of OGTT, (**D**) AUC of ITT, and serum of (**E**) TC, (**F**) TG, (**G**) LDL-C, and (**H**) HDL-C. (*), (**) and (***) represent *p* < 0.05, *p* < 0.01 and *p* < 0.001 compared with NC mice, and (#), (##) and (###) represent *p* < 0.05, *p* < 0.01 and *p* < 0.001 compared with TIIDM mice, respectively. The following abbreviations are used in this figure: OGTT (oral glucose tolerance test); ITT (insulin tolerance test); AUC of OGTT (Area Under the Curve of OGTT); AUC of ITT (Area Under the Curve of ITT); TC (total cholesterol); TG (triglycerides); LDL-C (low-density lipoprotein cholesterol); HDL-C (high-density lipoprotein cholesterol).

As shown in Figure 4A, following oral glucose gavage, the TIIDM mice exhibited a markedly higher increase and peak in blood glucose levels compared to the NC group, along with a prolonged duration of elevation. Similarly, the TIIDM group showed a significantly smaller reduction in blood glucose levels after intraperitoneal insulin injection compared with the NC mice (Figure 4B). Notably, FT treatment effectively suppressed the glucose spike following oral gavage and resulted in a more rapid response to insulin than observed in the untreated TIIDM group. Furthermore, compared with the NC mice, the Area Under the Curve (AUC) for both OGTT and ITT was significantly increased in TIIDM mice (Figure 4C,D, *p* < 0.001). While FT intervention successfully reduced the AUC of OGTT and ITT (*p* < 0.01), T treatment did not lead to a significant reduction in AUC values (*p* > 0.05). Collectively, these data indicated that TIIDM mice were characterized by impaired glucose tolerance and reduced insulin sensitivity, both of which were significantly improved through FT intervention.

As shown in Figure 4E,F, serum levels of TC and TG were significantly elevated in the TIIDM group compared to the NC group (*p* < 0.001). Compared with the TIIDM group, FT treatment (FTM and FTH) significantly reduced serum TC levels (*p* < 0.05, *p* < 0.01), although the magnitude of this reduction was less pronounced than that observed in the MET-treated TIIDM mice. Furthermore, a significant decrease in TC levels was also observed in the TH group relative to the TIIDM group (*p* < 0.01). As illustrated in Figure 4G,H, TIIDM mice exhibited markedly higher LDL-C levels and significantly lower HDL-C levels compared with the NC group (*p* < 0.001, *p* < 0.001). Following 6 weeks of oral FT intervention, serum LDL-C levels in TIIDM mice significantly decreased (*p* < 0.05, *p* < 0.001), while HDL-C levels significantly increased compared to the untreated TIIDM mice (*p* < 0.01, *p* < 0.01), but unfermented PMT treatment did not significantly improve the abnormal of blood lipids in TIIDM mice (*p* > 0.05). Collectively, these results demonstrated that EC-fermented PMT treatment effectively ameliorated both hyperglycemia and dyslipidemia in TIIDM mice.

### 3.5. Oral Administration of EC-Fermented PMT Alleviated the Visceral Damage in TIIDM Mice

**Figure 5:**
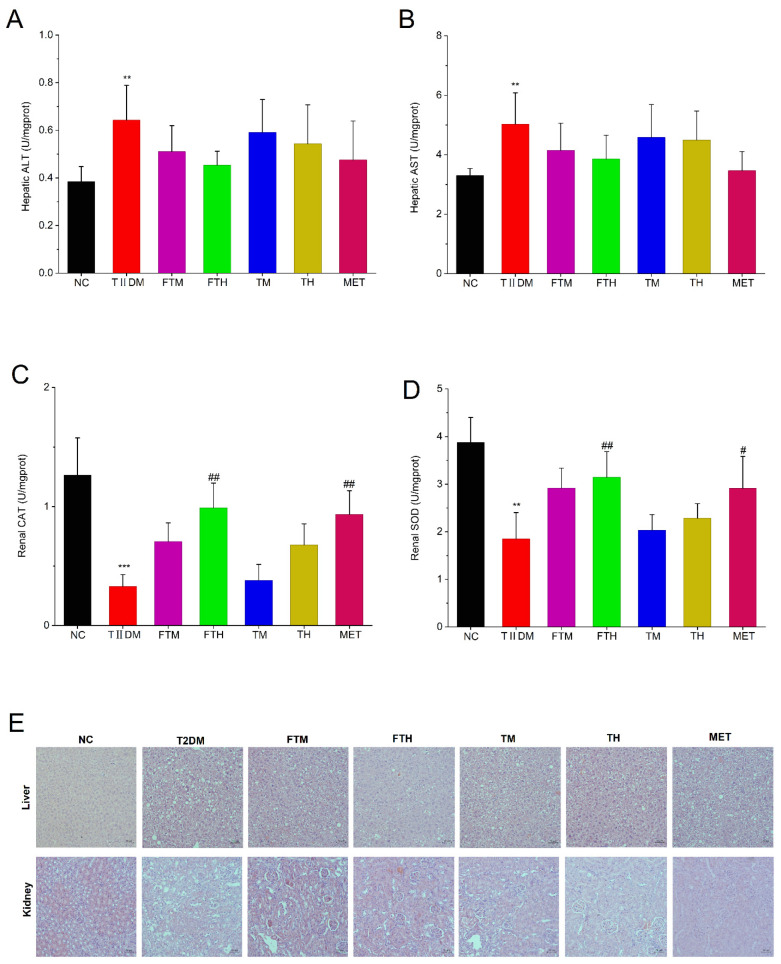
Oral administration of *Eurotium cristatum*-fermented *Premna microphylla* Turcz. alleviated the liver and kidney damage in TIIDM mice (*n* = 10). The levels of hepatic (**A**) ALT and (**B**) AST, renal (**C**) CAT and (**D**) SOD, and (**E**) hematoxylin–eosin staining of the liver and kidney. (*), (**) and (***) represent *p* < 0.05, *p* < 0.01 and *p* < 0.001 compared with NC mice, and (#), (##) and (###) represent *p* < 0.05, *p* < 0.01 and *p* < 0.001 compared with TIIDM mice, respectively.

As shown in Figure 5A,B, relative to the NC group’s mice, serum levels of ALT and AST in the TIIDM group’s mice were significantly elevated (*p* < 0.01). However, following oral administration of fermented PMT, both ALT and AST levels in TIIDM mice exhibited varying degrees of reduction, but no significance (*p* > 0.05). As shown in Figure 5C,D, compared with the NC group, the levels of CAT and SOD in the kidney of TIIDM mice were significantly lower (*p* < 0.001, *p* < 0.01). Intervention with high-dose EC-fermented PMT aqueous extract (FTH) markedly enhanced kidney CAT and SOD activities in TIIDM mice (*p* < 0.01). In contrast, unfermented PMT aqueous extract treatment failed to significantly increase CAT and SOD levels in TIIDM mice (*p* > 0.05).

Furthermore, histopathological examination revealed that the hepatocytes in the NC group were compactly arranged with relatively small volumes and homogeneously stained nuclei (Figure 5E). In contrast, extensive vacuolation was observed in the liver of the TIIDM group. Following FT intervention, the prevalence of hepatic vacuoles was markedly reduced. Regarding the kidney, the NC group exhibited a clear and intact renal architecture with tightly connected cells. Conversely, the TIIDM group showed loosely arranged cells with significant cellular swelling (hypertrophy), indicating evident renal injury. Notably, treatment with FT, T, and MET resulted in a slight alleviation and histological recovery of the observed renal damage.

**Figure 6:**
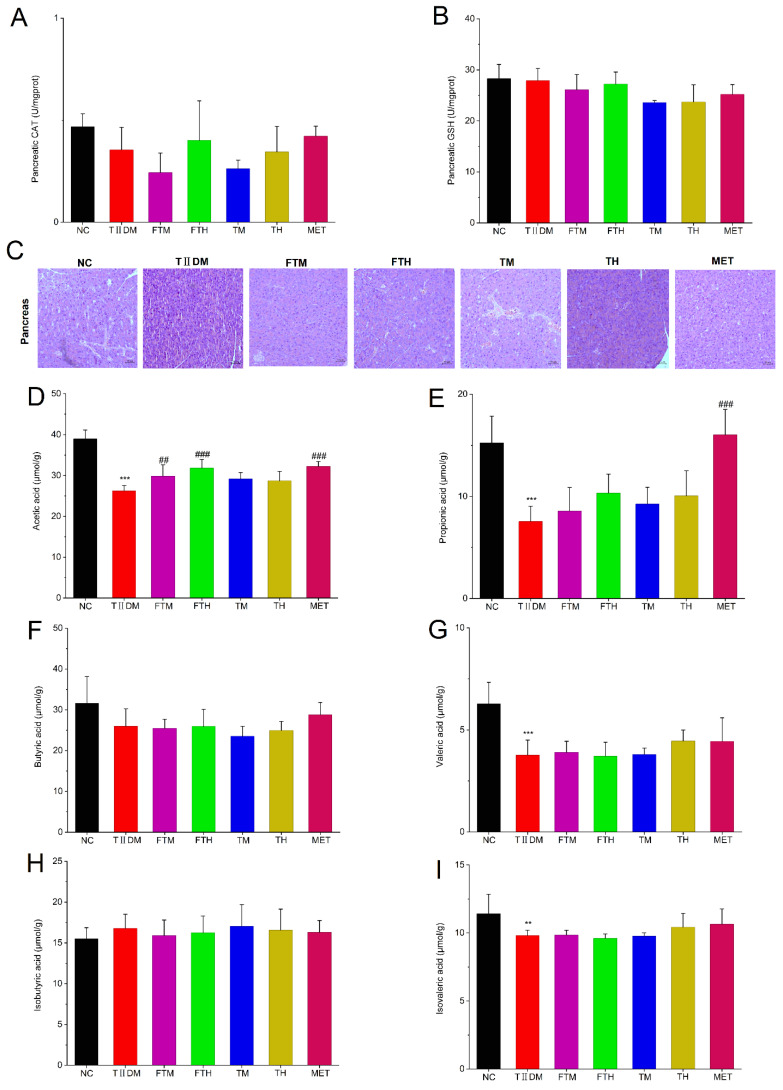
*Eurotium cristatum*-fermented *Premna microphylla* Turcz. promoted pancreatic damage and increased the production of short-chain fatty acids in TIIDM mice (*n* = 10). The contents of pancreatic (**A**) CAT and (**B**) GSH; (**C**) hematoxylin–eosin staining of the pancreas. The levels of (**D**) acetate, (**E**) propionate, (**F**) butyrate, (**G**) valerate, (**H**) isobutyrate and (**I**) isovalerate in cecal contents. (*), (**) and (***) represent *p* < 0.05, *p* < 0.01 and *p* < 0.001 compared with NC mice, and (#), (##) and (###) represent *p* < 0.05, *p* < 0.01 and *p* < 0.001 compared with TIIDM mice, respectively.

Compared with the NC mice, no significant downward trend was observed in pancreatic CAT activity or GSH levels in the TIIDM group (Figure 6A,B, *p* > 0.05). Likewise, no significant differences were identified following FT, T, or MET interventions (*p* > 0.05). H&E staining revealed that the pancreatic tissues of TIIDM mice exhibited increased intercellular gaps, diminished islet cluster sizes, and evidence of partial cell rupture (Figure 6C). In contrast, the pancreatic architecture in the FT and T intervention groups appeared more integrated, demonstrating a trend toward restored histological integrity and normal tissue morphology. Collectively, these findings demonstrated that hepatic, renal and pancreatic injuries in TIIDM mice were significantly ameliorated following intervention with EC-fermented PMT.

### 3.6. Supplementation of EC-Fermented PMT Increased the Contents of SCFAs in TIIDM Mice

Next, the levels of SCFAs in cecal contents were measured by GC-MS. As shown in Figure 6D,I, relative to the NC group, the concentrations of SCFAs in the cecal contents of the TIIDM group, specifically acetic acid, propionic acid, and valeric acid, were significantly reduced (*p* < 0.001). Conversely, intervention with EC-fermented PMT effectively elevated the levels of acetate in the cecal contents of TIIDM mice. Notably, MET treatment significantly elevated the levels of propionate. Overall, these data indicated that intervention with EC-fermented PMT can increase the levels of SCFAs in TIIDM mice.

## 4. Discussion

The present study provides the first evidence that fermentation by EC significantly enriches the nutritional profile and enhances the antidiabetic potential of PMT. Fermentation led to a marked increase in pectin, soluble polysaccharides, flavonoids, and specific short-chain fatty acids, including butyric and valeric acids, while reducing the levels of polyphenols and soluble proteins. In vivo results demonstrated that EC-fermented PMT effectively alleviated hyperglycemia-induced organ damage, which was closely associated with improved glucose tolerance and enhanced insulin sensitivity in TIIDM mice.

The observed changes in nutritional composition can be largely attributed to the metabolic activities of EC during fermentation [27]. As a representative probiotic fungus, EC secretes a variety of extracellular enzymes, including cellulases, polyphenol oxidases, and proteases, which facilitate the biotransformation of macromolecular components [17]. In the present study, the significant increase in pectin and soluble polysaccharides may result from the enzymatic degradation of insoluble dietary fibers, such as cellulose and lignin, into more bioavailable forms [28]. Additionally, extracellular polysaccharides produced by EC during growth may contribute to the overall polysaccharide content [26]. Monosaccharide composition analysis revealed that glucose and galacturonic acid remained the dominant constituents, suggesting that the fermented PMT polysaccharides may possess potential prebiotic properties [29,30]. In contrast, the reduction in soluble proteins is likely due to their utilization as nitrogen sources to support microbial growth, which also led to alterations in amino acid composition. The decrease in polyphenol content may be explained by their enzymatic oxidation into polymerized pigments, contributing to the observed color changes after fermentation [17,31]. Similarly, the levels of polyphenols and catechins in summer and autumn teas decrease significantly after EC fermentation [32]. Notably, the increase in flavonoid content suggests that EC fermentation enhances the release or transformation of bound flavonoids, thereby improving their bioaccessibility [33]. After fermentation via EC, the glycoside isoflavones in green kernel black beans are converted into saponinic isoflavones [34]. A previous study has also shown that flavonoids extracted from Mao Jian green tea regulate AMPK expression, inhibit hepatic gluconeogenesis, and improve glucose metabolism in type2-diabetic rats [35]. However, the specific flavonoid compounds responsible for these effects were not identified, and the hepatic gluconeogenesis pathway was not analyzed in this study, and it warrants further investigation.

The antidiabetic effects of EC-fermented PMT were further validated in vivo using an HFD/STZ-induced TIIDM mouse model. Compared with non-fermented PMT, the fermented product exhibited superior dose-dependent hypoglycemic activity, as evidenced by improved glucose tolerance and insulin sensitivity. Moreover, EC-fermented PMT significantly ameliorated dyslipidemia, as indicated by decreased serum levels of TC, TG, and LDL-C, along with increased HDL-C. These findings suggest that EC fermentation enhances the metabolic regulatory functions of PMT. Similarly, EC-fermented Coptis herbal pairs can improve symptoms in rats with TIIDM by promoting the proliferation of beneficial gut bacteria such as *Ligilactobacillus* and *Akkermansia*, thereby regulating glucose and lipid metabolism [20]. Chronic consumption of a high-fat diet is known to induce hepatic and renal dysfunction through oxidative stress and inflammatory responses [36]. In agreement with this, TIIDM mice in the present study exhibited elevated levels of ALT and AST, along with reduced antioxidant enzyme activities, such as CAT and SOD. Notably, administration of EC-fermented PMT effectively reversed these pathological alterations, indicating its protective effects against oxidative damage and tissue injury.

SCFAs are key microbial metabolites that play essential roles in maintaining host metabolic homeostasis [37]. Reduced SCFA levels are commonly observed in TIIDM and are closely associated with insulin resistance [38]. Non-digestible polysaccharides serve as a carbon source for the gut microbiota, and the primary metabolites produced by the gut microbiota are SCFAs. In the present study, EC-fermented PMT significantly increased the levels of SCFAs, particularly acetic acid, in the cecal contents of TIIDM mice. It is likely that such effects resulted from a marked increase in soluble polysaccharides following EC fermentation of PMT. Acetic acid has been reported to modulate the gut environment by lowering intestinal pH, inhibiting pathogenic bacteria, and promoting the growth of beneficial microbiota [39]. Furthermore, it can stimulate the secretion of gut hormones such as glucagon-like peptide-1 (GLP-1) and peptide YY (PYY), thereby contributing to improved glucose metabolism and energy balance [40,41]. These findings suggest that the antidiabetic effects of EC-fermented PMT may be partially mediated through SCFA-associated mechanisms. However, we did not investigate the effects of EFPT on the composition and abundance of the gut microbiota in TIIDM mice, and further exploration is warranted to elucidate the antidiabetic mechanism of EFPT.

In summary, EC fermentation significantly enhances the nutritional and functional properties of PMT by promoting the transformation of bioactive components and increasing SCFA production. The fermented product exhibits potent antidiabetic effects, including improved glucose and lipid metabolism and protection against organ damage in TIIDM mice. These findings provide new insights into the development of PMT-based functional foods and highlight the potential application of EC in the production of value-added fermented products. However, the current data are still limited to mammalian models. Future research will require clinical trials to confirm the efficacy and safety of EC-fermented foods.

## Figures and Tables

**Table 1:** The monosaccharide composition of polysaccharide extract from *Eurotium cristatum*-fermented *Premna microphylla* Turcz. (EFPT).

Time (Day)	0	2	4	6	8
Monosaccharide composition (molar %)					
Mannose	1.04	0.60	0.68	1.19	1.17
Ribose	N.D.	N.D.	N.D.	N.D.	N.D.
Rhamnose	17.19	12.10	12.69	13.56	18.84
Glucuronic acid	17.28	21.78	24.93	28.70	14.14
Galacturonic acid	N.D.	N.D.	N.D.	N.D.	N.D.
Glucose	46.51	51.40	47.46	38.01	41.67
Galactose	9.92	9.15	9.12	11.79	14.55
Arabinose	7.06	4.96	5.12	6.74	9.63

N.D. = not detected.

## Data Availability

The original contributions presented in this study are included in the article. Further inquiries can be directed to the corresponding authors.
